# Benzylpyrazinium Salts as Photo-Initiators in the Polymerization of Epoxide Monomers

**DOI:** 10.3390/ma7085581

**Published:** 2014-07-31

**Authors:** Moon Suk Kim, Sang Bong Lee

**Affiliations:** 1 Department of Molecular Science and Technology, Ajou University, Suwon 443-749, Korea; E-Mail: moonskim@ajou.ac.kr; 2 Division of Green Chemistry & Engineering Research, Research Institute of Chemical Technology, Daejeon 305-600, Korea

**Keywords:** ultraviolet (UV) light irradiation, photo-initiators, latent, epoxide monomers, benzyl pyrazinium salt

## Abstract

In order to study the capability of pyrazinium salt derivatives to act as photo-initiators of epoxide monomers, benzyl pyrazinium hexafluoroantimonate (BPH), benzyl 3,5-dimethyl pyrazine hexafluoroantimonate (BDH) and benzyl quinoxalinium hexafluoroantimonate (BQH) were synthesized by the Menschutkin reaction of benzyl bromide with pyrazine, 2,6-dimethyl pyrazine, and quinoxaline, followed by exchanging with hexafluoroantimonate (SbF_6_). BPH, BDH, and BQH exhibited characteristic ultraviolet (UV) absorbance as well as exothermic peaks as a function of irradiation time in a differential photo-calorimeter (DPC). In the absence of photo-irradiation, cyclohexene oxide (CHO) underwent slow polymerization at 25 °C using BPH derivatives, but quantitative conversion was achieved even after a 5-min photo-irradiation. In addition, photo-irradiation was required for the photo-polymerization of CHO and styrene oxide (STO), which was characterized by a short induction period followed by a very rapid and exothermic polymerization. While glycidyl methyl ether (GME) required long induction periods, glycidyl phenyl ether (GPE) underwent rather slow and/or no photo-polymerization. The reactivity order of the monomers was CHO > STO >> GME >>> GPE, and the reactivity order for the photo-polymerization of CHO was BPH > BQH > BDH. It was found that BPH, BDH, and BQH could serve as photo-latent initiators for CHO, STO and GME, respectively.

## 1. Introduction

Initiators are chemicals that can produce active species under normal or special conditions. Among the several initiators available, “latent initiators” show no activity under normal conditions, but form active species under certain external stimulations such as heating and photo-irradiation [1]. Latent initiation is one of the most promising candidates for controlling polymerization.

Meanwhile, the development of one-component systems with premixed monomers/resins and latent initiators offer excellent processability in practical industrial applications such as paints, inks, and adhesives [2,3,4,5,6,7,8]. Ideally, such one-component systems may be stored as a one-component product for a certain period prior to use and they can initiate or cure polymerization only through targeted external stimulation. Therefore, latent initiators can be practically utilized in industries.

Among the several external stimulations, photo-irradiation can initiate rapid polymerization (curing), making it a highly attractive external stimulant compared to the complex and energy-consuming thermal polymerization [9]. In addition, photo-latent initiators can enhance the pot life of one-component systems, thereby reducing the time taken for polymerization.

Several groups have developed several onium salt series as photo-cationic initiators for the cationic polymerization of cyclic ethers, alkyl vinyl ethers, and epoxides [10,11,12,13,14,15]. The sulfonium and iodonium salts were indefinitely stable without photo-irradiation, but under photo-irradiation, initiating species were generated, which polymerized rapidly without inhibition or retardation, indicating the presence of a latent initiator. Some commercial grade initiators have also been developed.

Recently, we developed benzyl pyrazinium hexafluoroantimonate (BPH), which is a thermal cationic initiator [16,17]. While studying the mechanism of benzylpyrazinium salts (BPS) under thermal stimulation, BPH generated a benzyl cation as the actual initiation species and pyrazine as the polymerization terminator. The formed benzyl cation was attacked by monomers such as epoxides, which subsequently formed the rapidly propagating cationic species for monomer polymerization. At the end of the propagation step, the low concentration monomer competed with pyrazine to terminate the polymerization. Hence, BPH successfully served as the thermal latent initiator in epoxide polymerization.

**Figure 1:**
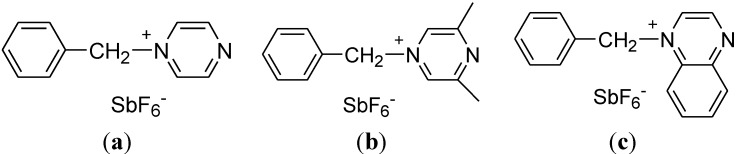
Structures of: (**a**) benzyl pyrazinium hexafluoroantimonate (BPH); (**b**) benzyl 3,5-dimethyl pyrazine hexafluoroantimonate (BDH); and (**c**) benzyl quinoxalinium hexafluoroantimonate (BQH). SbF_6_: hexafluoroantimonate.

To our knowledge, however, BPH has received less attention as a photo-latent initiator despite possessing specific ultraviolet (UV) absorbance due to its unique molecular structure. Thus, the aim of this study was to examine the potential of BPH, benzyl 3,5-dimethyl pyrazine hexafluoroantimonate (BDH) and benzyl quinoxalinium hexafluoroantimonate (BQH) as photo-latent initiators (Figure 1). In this study, we investigated the following issues: (1) reaction of BPH, BDH, and BQH under photo-irradiation; (2) comparison of the activation of BPH, BDH, and BQH under photo-irradiation and thermal stimulation; and (3) effect of BPH, BDH, and BQH as photo-latent initiators. Elucidation of these issues will have an important impact on the novel applications of BPH derivatives as photo-latent initiators.

## 2. Experimental Section

### 2.1. Materials

Benzyl bromide (98%), pyrazine (99%), 2,6-dimethyl pyrazine (99%), quinoxaline (99%), and sodium hexafluoroantimonate (NaSbF_6_, technical purity grade) were purchased from Aldrich (Milwaukee, WI, USA) and used as received without further purification. Cyclohexene oxide (CHO), styrene oxide (STO), glycidyl methyl ether (GME), and glycidyl phenyl ether (GPE) were purchased from Aldrich and distilled over calcium hydride before use.

### 2.2. Measurements

^1^H nuclear magnetic resonance (NMR) spectra were recorded with a SYKES disk 7000 Varian 300 MHz FT/NMR spectrometer (Santa Clara, CA, USA) using tetramethylsilane (TMS) as the internal standard in acetone-d_6_. Infrared (IR) spectra were measured using a JASCO FT/IR-3 spectrophotometer (Tokyo, Japan). Melting points (MP) were measured by a Thomas-Hoover capillary melting point apparatus (Philadelphia, PA, USA). Number- and weight-average molecular weights (*M*_n_ and *M*_w_), and polydispersity ratios (*M*_w_/*M*_n_) were estimated by gel permeation chromatography (GPC) on a Waters 2690 with a refractive index (RI) detector (Waters 410RI, Milford, MA, USA). The GPC was equipped with two consecutive polystyrene (PS) gel columns (PLgel, 5 μm, 500 Å (500–30,000: molecular weight of exclusion limit); and PLgel mixed B, 10 μm (500,000–10,000,000: molecular weight of exclusion limit)) at 40 °C. Tetrahydrofuran (THF) was used as the eluent with a flow rate of 1.0 mL/min by PS calibration. Elemental analyses were carried out with a Perkin-Elmer 240C CHN (Waltham, MA, USA).

### 2.3. Synthesis of BPH and BDH

BPH and BDH were prepared according to a previously reported method [16].

### 2.4. Synthesis of BQH

Quinoxaline (50 g, 0.38 mol) was added to benzyl bromide (242 g, 1.42 mol) at room temperature and the mixture was stirred for 48 h. The precipitate was filtered and washed several times with benzene. An aqueous solution of NaSbF_6_ (88.5 g, 0.34 mol) was added to the solution of the resulting solid. After stirring for 5 min, a white precipitate was formed, which was filtered and washed several times with ether. It was crystallized from CH_3_OH to give 253.2 g (0.34 mol, 89.5%) of a white crystal; MP 147.8–151.2 °C. IR (KBr, cm^−1^): 3098, 1514, 1359, 1073, 767, 667 (hexafluoroantimonate, SbF_6_). ^1^H NMR (acetone-d_6_): 8.66 (s, 1H, CHN^+^), 8.46 (s, 1H, CHN), 8.13 (m, 5H, C_6_H_5_), 7.60 (m, 4H, –C_6_H_4_), 6.26 (s, 2H, –CH_2_). Analytic calculation for C_15_H_13_N_2_SbF_6_: C, 39.42; H, 2.87; N, 6.13. Found: C, 39.69; H, 2.86; N, 6.16.

### 2.5. Differential Photo-Calorimeter (DPC)

A DPC (Model 930, TA Instruments, Wilmington, DE, USA) was used to monitor the isothermal heat generated during the UV irradiation at 30 °C. A 200 W Hg arc lamp was used as the UV source. BPH, BDH, and BQH (1.5 ± 0.1 mg) were set up in standard Du Pont Al pans and stabilized for 15 min in nitrogen atmosphere in the DPC sample chamber prior to UV exposure. The heat flow was monitored as a function of irradiation time.

### 2.6. Polymerization

Typical procedure for CHO epoxide polymerization is as follows: BPH (24.4 mg, 0.06 mmol) was taken in a pyrex glass tube. In this work, the polymerization was accomplished by using only 3 mol% of BPH derivatives according to previously reported results [12,13,16]. In order to remove oxygen, the reaction tube was flushed with nitrogen and sealed with a septum. Under nitrogen, CHO (301 mg, 2 mmol) was fed into the tube with a syringe. Photo-irradiation was conducted with a Hanovia 450 W medium-pressure Hg arc lamp (5.0 mW/cm^2^, λ_max_ = 262 nm, λ_max_ = 313 nm). The tube was subsequently placed in a rotating “merry-go-round” holder to provide even illumination throughout the photo-irradiation process. The Hg arc lamp was at a distance of 15 cm from the reaction tube. The entire apparatus was placed in a large thermostated water bath which controls the temperature within 1 °C. After a certain period of time, one drop of 5% NH_4_OH was added to the tube. The mixture was then poured into 50 mL CH_3_OH to precipitate the polymer. The polymer was separated from the supernatant by decantation and dried *in vacuo*. Prior to precipitation with CH_3_OH, the monomer conversion was determined by ^1^H NMR spectroscopy and the molecular weight of the polymer was determined by GPC. The obtained polymer was identified to be poly(CHO).

## 3. Results and Discussion

### 3.1. Preparation and Characterization of BPH, BDH and BQH

BPH, BDH and BQH used in this study were prepared by the Menschutkin reaction of benzyl bromide with pyrazine, 2,6-dimethyl pyrazine, and quinoxaline [16]. Due to less solubility in benzyl bromide, the corresponding benzyl quaternary ammonium bromides were obtained as white precipitates. In the second reaction step, the bromide ion of benzyl pyrazinium bromide, benzyl-3,5-dimethylpyrazinium bromide and benzyl quinoxalinium bromide were exchanged with SbF_6_.

The structures of BPH, BDH, and BQH were confirmed by ^1^H NMR, IR spectroscopy, and elemental analysis. Only the monoquarternized structure of one nitrogen atom in pyrazine was identified by ^1^H NMR and elemental analysis, probably due to the drastic decrease in nucleophilicity of the other nitrogen atom after monoquarternization. In the IR spectrum, the characteristic signal of SbF_6_, as the counteranion of pyrazinium salts, appeared at 660 cm^−1^.

**Figure 2:**
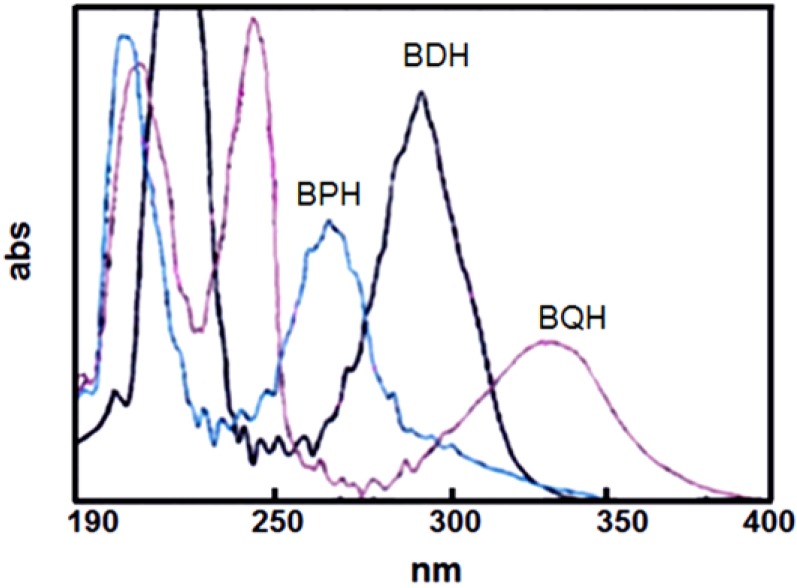
Ultraviolet (UV) spectra of BPH, BDH and BQH.

As shown in Figure 2, UV absorptivities were studied by measuring the UV absorbances of BPH, BDH and BQH. The absorption spectra of BPH, BDH, and BQH ranged from 250 nm to 500 nm, and the absorption maxima was observed around 270 nm, which was similar to that of a Hg arc lamp.

**Figure 3:**
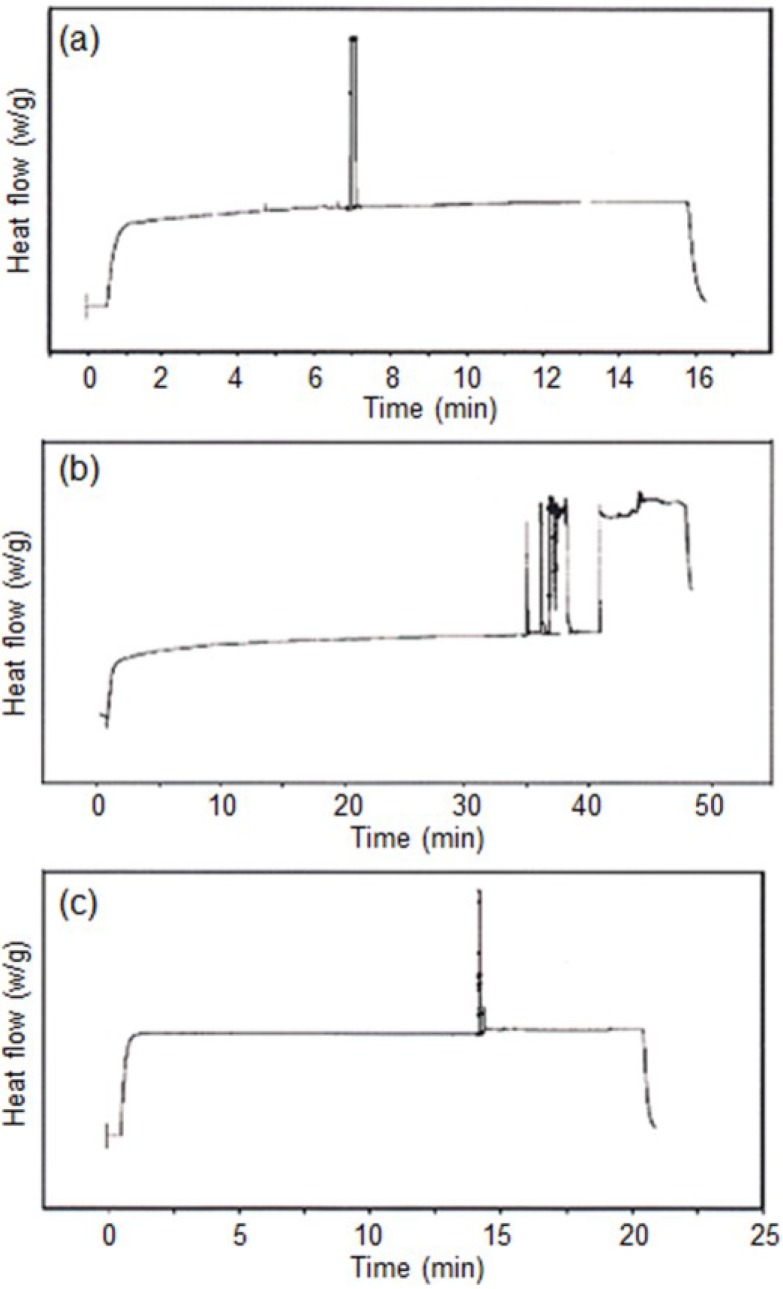
Differential photo-calorimeter (DPC) curves for: (**a**) BPH; (**b**) BDH; and (**c**) BQH under photo-irradiation.

Figure 3 shows the heat flow of BPH, BDH, and BQH as a function of irradiation time. The exothermic peaks of BPH, BDH, and BQH were observed at 7 min, 35 min, and 14 min, respectively. This clearly indicated the photo-activation of BPH, BDH, and BQH. Thus, BPH, BDH, and BQH may be activated by photo-irradiation and applied in the photo-polymerization of epoxide monomers.

### 3.2. Photo-Polymerization

We aimed to study the capability of BPH, BDH, and BQH to initiate the photo-polymerization of epoxide monomers. Therefore, we first chose the CHO monomer that is prone to undergo cationic polymerization.

**Figure 4:**
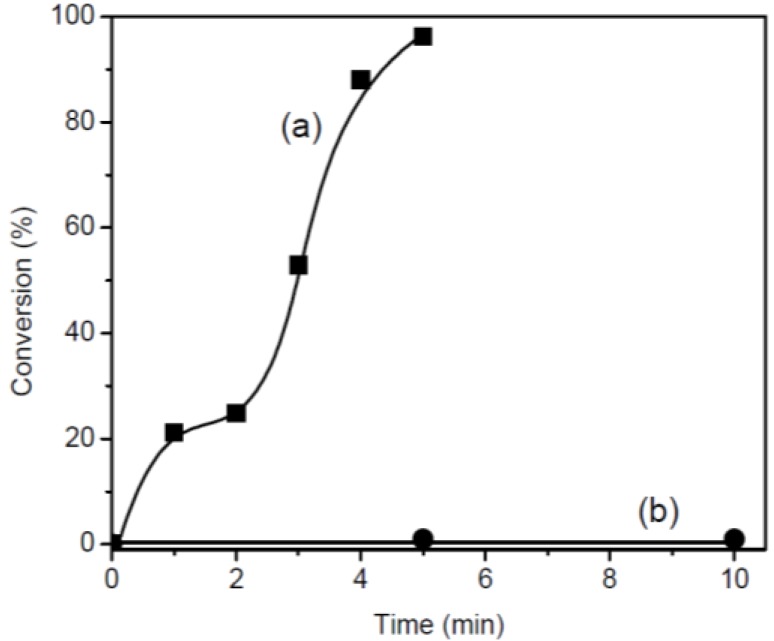
Time-conversion curves for photo-polymerization of cyclohexene oxide (CHO) in the presence of 3 mol% BPH: (**a**) with and (**b**) without photo-irradiation at 25 °C.

CHO was polymerized in bulk using 3 mol% of BPH at 25 °C for 30 min without photo-irradiation and 10 min with photo-irradiation (Figure 4). The polymerization proceeded homogeneously throughout the reaction because BPH was completely soluble in CHO at 25 °C. Limited polymerization of CHO took place with BPH at 25 °C. In the absence of photo-irradiation, only 1%–2% conversion of CHO was observed even after 30 min, indicating that the polymerization takes place slowly at 25 °C without photo-irradiation. Meanwhile, the photo-irradiation of CHO exhibited almost quantitative conversion even at 5 min. This indicated that BPH could induce the polymerization of CHO under photo-irradiation.

The effect of photo-irradiation time on the conversion of CHO exhibited sigmoidal curves. We investigated the sigmoidal curves by measuring the reaction heat based on photo-irradiation. When a non-polymerizing blank (CHO without a BPH) was irradiated for 30 min, no changes in temperature (25 °C) were observed due to UV absorption. In the absence of photo-irradiation, the CHO-BPH mixture also maintained a temperature of 25 °C for 30 min. Upon photo-irradiation, temperature of the CHO-BPH mixture increased very slowly from 25 °C to 30 °C over 2 min. Then, after 4 min of photo-irradiation, the temperature of the mixture reached approximately 65 °C. Thus, it can be concluded that the observed rise in temperature is due to an exothermic reaction that occurred during polymerization.

This implied that the photo-polymerization of CHO could be accelerated by the exothermic reaction heat, which was caused by photo-irradiation [18]. Thus, the photo-polymerization of CHO was characterized by an induction period followed by a very rapid and exothermic polymerization. Therefore, as a result of the heat produced by the exothermic reaction, the photo-irradiation time-conversion of CHO exhibited sigmoidal curves.

**Figure 5:**
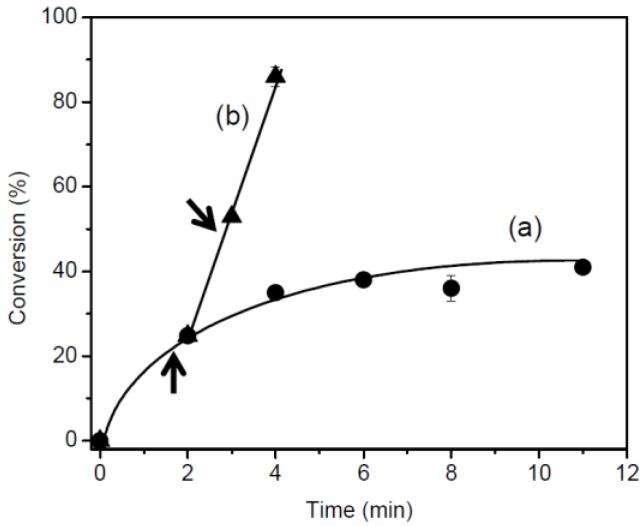
Time-conversion curves for photo-polymerization of CHO in the presence of BPH (3 mol%) at turn off at: (**a**) 1 min 55 s; and (**b**) 2 min 55 s. The arrows indicated the time of turn off.

The effect of photo-irradiation was examined by stopping the polymerization of CHO after 1 min 55 s and 2 min 55 s (Figure 5). When the reaction was stopped at 1 min 55 s, CHO exhibited 24% conversion at 2 min and 39% conversion at 11 min. Stopping at 2 min 55 s resulted in 52% conversion at 3 min and 88% at 4 min. This indicated that extended photo-irradiation was required for the photo-polymerization of CHO.

**Figure 6:**
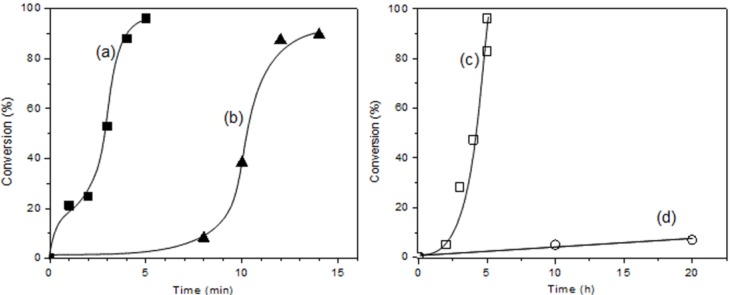
Time-conversion curves for the photo-polymerization of: (**a**) CHO; (**b**) styrene oxide (STO); (**c**) glycidyl methyl ether (GME); and (**d**) glycidyl phenyl ether (GPE) carried out in bulk with 3 mol% BPH.

We performed the photo-polymerization of STO, GME, and GPE, for comparing their monomer activities with BPH under photo-irradiation (Figure 6). Each monomer displays a different and characteristic photo-irradiation time-conversion response. Photo-polymerization of CHO and GME exhibited short induction periods, which resulted in a very high reaction rate. The high reactivity of CHO is due to the additional ring strain that results from the incorporation of the epoxy group into a bicyclic group. The high reactivity of STO is probably due to induction effect of phenyl group.

Meanwhile, the photo-polymerization of GME showed above 80% conversion at 5 h of photo-irradiation. GME displayed a long induction period of 2 h during which minimal reaction took place, followed by polymerization. BPH polymerized only 7% of GPE even at 20 h. GPE underwent no photo-polymerization without a substantial induction period.

Table 1 shows the molecular weight of the polymers obtained with BPH. Although STO and GME reached quantitative conversion, oligomers were formed, probably due to several side reactions such as chain transfer and formation of cyclic oligomers. This is due to the back-biting reaction, characteristic of epoxide monomers [1]. Meanwhile, increase in CHO conversion resulted in an increase in the molecular weight of poly(CHO) with photo-irradiation time. However, the molecular weight of poly(CHO) slightly decreased at high CHO conversion due to the back-biting reaction. In this study, CHO and STO showed high photo-polymerization rates whereas GME exhibited long induction periods and slow polymerization rates.

**Table 1:** Molecular weight of the polymers obtained from the photo-polymerization of CHO with BPH.

Monomer	Time (min)	Conversion (%)	*M*_n_	*M*_w_/*M*_n_
CHO	2	25	4180	2.86
	3	53	5920	2.61
	4	88	7460	2.49
	5	96	7000	2.76
STO	8	trace	-	-
	10	38	-	-
	12	87	900	1.28
	14	90	730	1.26
GME	2	trace	-	-
	3	28	840	1.38
	4	47	760	1.27
	5	83	810	1.40

**Figure 7:**
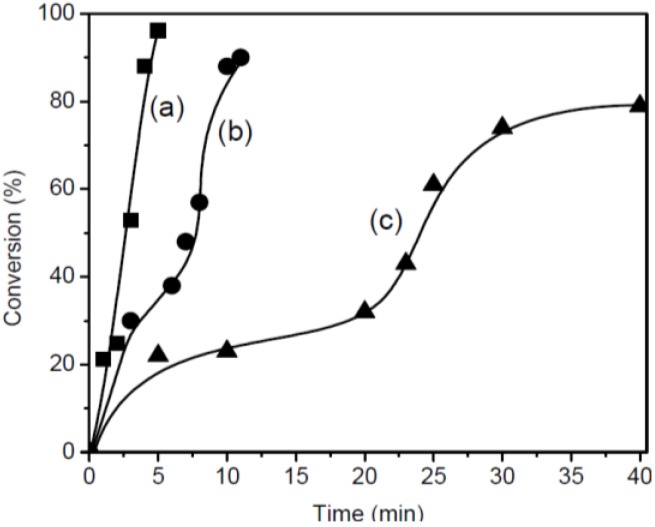
Time-conversion curves for the photo-polymerization of CHO carried out in bulk with 3 mol% salt: (**a**) BPH; (**b**) BQH; and (**c**) BDH as the photo-initiator.

CHO was photo-polymerized in bulk using BPH, BDH, and BQH. The polymerization proceeded homogeneously throughout the reaction because BPH, BDH, and BQH were completely soluble in CHO at ambient temperature. Figure 7 shows the photo-irradiation time-conversion curves of polymerization. The reactivity order was BPH > BQH > BDH. This order was in agreement with the order of heat flow of BPH, BDH, and BQH in DPC. The photo-irradiation time-conversion of the polymerization also exhibited sigmoidal curves.

## 4. Conclusions

In this work, we examined the potential of BPH derivatives as photo-latent initiators. Compared to thermal reactions, BPH, BDH, and BQH showed effective polymerization under photo-irradiation. Thus, the BPH derivatives successfully served as photo-latent initiators in the polymerization of CHO, STO and GME. This photoactive system could be used as an epoxy resin hardener. Research on the molecular design for other BPH derivatives and the reactions using epoxy resin is now underway and will be reported in the future.
